# Solitary brain metastasis from laryngeal squamous cell carcinoma in a 68-year-old male: A Case Report

**DOI:** 10.1097/MS9.0000000000003549

**Published:** 2025-07-18

**Authors:** Allahdad Khan, Abdul Ahad Riaz, Shahroze Ahmed, Anam Malik, Mohamed Antar, Raheel Ahmed

**Affiliations:** a Department of Medicine, Nishtar Medical University, Multan, Pakistan; b Department of Radiology, Shaukat Khanum Memorial Cancer Hospital & Research Centre, Lahore, Pakistan; c Department of Pathology, Nishtar Medical University, Multan, Pakistan; d Faculty of Medicine, Tishreen University Faculty of Medicine, Latakia, Syrian Arab Republic; e National Heart and Lung Institute, Imperial College London, London, UK

## Abstract

**Introduction and importance:**

Laryngeal squamous cell carcinoma (LSCC) commonly metastasizes to regional lymph nodes, lungs, liver, and bones. Intracranial metastasis from LSCC is exceedingly rare, reported in only 0.4% of cases. The atypical presentation can delay diagnosis and negatively impact prognosis. We report a rare case of solitary brain metastasis from LSCC, highlighting the diagnostic challenges and clinical considerations.

**Case presentation:**

A 68-year-old Pakistani male with a prior diagnosis of LSCC presented with new-onset generalized tonic-clonic seizures. He had previously undergone total laryngectomy, radiotherapy, and chemotherapy. Brain MRI revealed a right frontal lobe lesion with surrounding edema, consistent with a solitary metastasis. Histopathology following craniotomy confirmed metastatic squamous cell carcinoma. The patient was managed with antiepileptics and referred for palliative whole-brain radiotherapy. He and his family opted for palliative care, declining further aggressive treatment.

**Clinical discussion:**

Distant brain metastasis in LSCC is rare and may occur without prior systemic spread. The mechanism may involve perineural invasion, although the exact pathophysiology remains unclear. Current diagnostic approaches include MRI and FDG-PET/CT. Due to limited cases, standardized treatment protocols are lacking. Management options include surgery, radiotherapy, chemotherapy, and palliative care, depending on disease progression and patient preference. Seizures as a presenting symptom are uncommon but may indicate intracranial involvement.

**Conclusion:**

This case emphasizes the need for high clinical suspicion and comprehensive neurological assessment in patients with advanced LSCC. Early diagnosis and multidisciplinary management are essential for improving outcomes in this rare but serious manifestation.

## Introduction

Laryngeal squamous cell carcinoma (LSCC) is one of the most common head and neck cancers, with a predilection for metastasizing to regional cervical lymph nodes, lungs, liver, and bones^[^1,2^]^. Distant metastasis is reported in approximately 1–4% of cases, with the lungs being the most frequent site, followed by bone and liver^[2]^. Intracranial metastasis from LSCC is extraordinarily rare, with few documented cases in the literature and is often discovered only after neurological symptoms emerge^[^2,3^]^. Pakistan has one of the highest global ASMRs for laryngeal cancer. In 2019, it stood at about 5.75 deaths per 100 000 population, the highest worldwide that year. In 2021, approximately 5617 deaths due to laryngeal cancer occurred in Pakistan^[^4,5^]^. The diagnostic challenge is heightened by its atypical presentation, which may delay timely intervention and influence the overall prognosis. This case report of a 68-year-old male with intracranial metastasis from LSCC aims to fill an existing gap by highlighting the critical need for heightened clinical vigilance and comprehensive neurological evaluation in advanced laryngeal cancers. This case report has been reported according to the SCARE 2025 guidelines^[6]^.

## Case presentation

A 68-year-old male of Pakistani origin with a known history of laryngeal squamous cell carcinoma (LSCC), diagnosed 1 year prior, presented to the emergency department with new-onset generalized tonic-clonic seizures. His initial oncologic management included a total laryngectomy followed by adjuvant radiotherapy and systemic chemotherapy. The patient had demonstrated initial tumor control. However, over the preceding weeks, he developed progressively worsening headaches, cognitive impairment, and intermittent episodes of confusion, ultimately culminating in multiple generalized seizures.

On examination, the patient was drowsy but arousable, with a Glasgow Coma Scale score of 12/15. No focal neurological deficits were noted. Vital signs were within normal limits, and there was no clinical evidence of systemic infection or metabolic disturbance. Examination of the laryngectomy stoma revealed it to be clean, patent, and free of signs of infection or obstruction.

**Figure 1.**
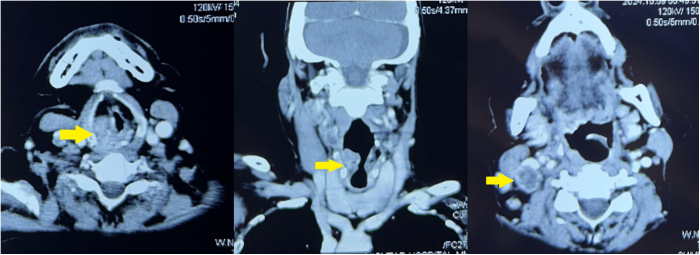
CT neck demonstrates a well-defined heterogeneous mass centered on the right true vocal cord with supraglottic extension. It is causing partial obstruction of the airway. There is associated cervical lymphadenopathy.

**Figure 2.**
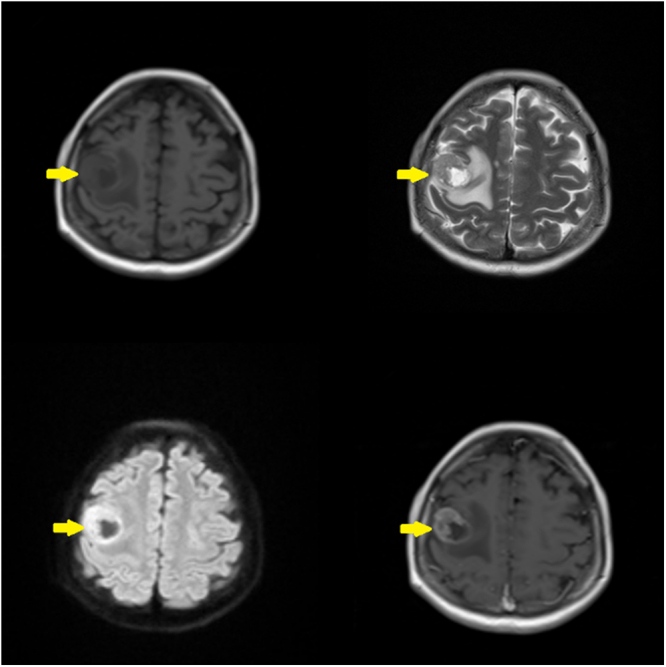
MRI brain is heterogeneous with an iso to hyper appearance in T2 and necrotic part visible in T1 post contrast. T1 hypointense, T2 hyperintense intraparenchymal lesion in the right premotor area of frontal lobe with peripheral post-contrast enhancement and extensive vasogenic edema. Blooming artifact is noted on GRE sequence, signifying internal hemorrhage. Findings are in keeping with a solitary metastatic lesion.

Electroencephalogram confirmed generalized epileptiform activity. Routine laboratory investigations were largely unremarkable, aside from mild normocytic anemia. CT scan of neck (Fig. 1) demonstrated a well-defined heterogeneous mass centered on the right true vocal cord with supraglottic extension. It is causing partial obstruction of the airway. There was associated cervical lymphadenopathy present. MRI brain (Fig. 2) shows a T1 hypointense, T2 hyperintense intraparenchymal lesion in the right frontal lobe with peripheral post-contrast enhancement and extensive vasogenic edema. Blooming artifact is noted on GRE sequence, signifying internal hemorrhage. Findings are in keeping with a solitary metastatic lesion.

**Figure 3.**
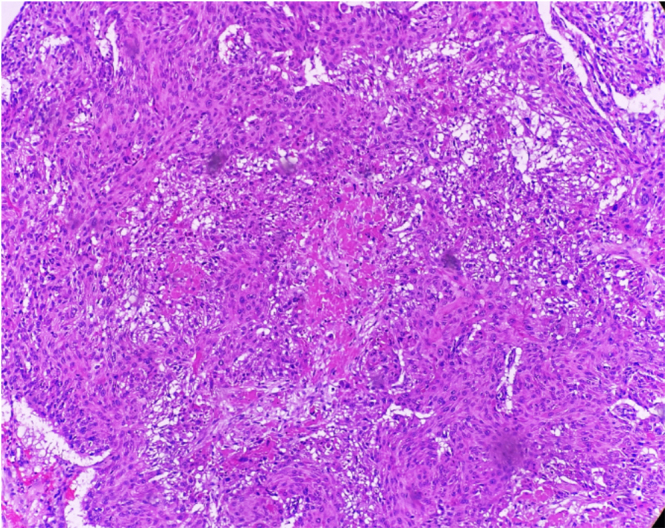
Neoplastic cells having abundant cytoplasm, atypical nuclei, and keratinization at the center.

A craniotomy was performed, and histopathological examination of the brain lesion confirmed squamous cell carcinoma (SCC), consistent with the primary laryngeal tumor (Fig. 3). He was started on intravenous levetiracetam for seizure control. A multidisciplinary oncology team was consulted, and palliative whole-brain radiotherapy was considered for symptomatic relief. Further systemic treatment options, including targeted therapy and immunotherapy, were discussed in the context of disease progression. After thorough discussion, the patient and his family opted for palliative care and declined therapeutic options such as gamma knife radiosurgery. The patient’s neurological condition stabilized with medical management, and he was discharged on oral antiepileptic therapy, with arrangements for close outpatient oncology follow-up and continuation of palliative care.

At his 3-month follow-up, the patient remained mostly seizure free on levetiracetam, though mild stress-induced seizure episodes occasionally occurred. Cognition had improved, with no focal deficits. He reported mild headaches, fatigue, and an unintentional 3 kg weight loss. Examination showed a clean, patent laryngectomy stoma. He continues on antiepileptic therapy with regular palliative care follow-up focused on symptom control.

**HIGHLIGHTS**

- Solitary brain metastasis from laryngeal squamous cell carcinoma (LSCC) is extremely rare, with few cases reported in the literature.
- The patient presented with new-onset generalized seizures, an uncommon initial symptom of LSCC metastasis.
- MRI findings and histopathological confirmation identified a solitary metastatic lesion in the frontal lobe, consistent with the primary laryngeal tumor.
- Perineural invasion may play a role in distant brain metastasis, despite the typical lymphatic spread of head and neck cancers.
- This case underscores the importance of neurological assessment and imaging in advanced LSCC, particularly when unexplained neurological symptoms occur.
- Due to the rarity of such cases, standardized treatment guidelines are lacking, and management often relies on individualized, multidisciplinary approaches.

## Discussion

SCC accounts for over 90% of cancers that develop in the larynx^[7]^.

Intracranial metastatic deposits from SCC of the larynx are exceedingly rare occurring approximately in 0.4% cases and usually are detected after development of pulmonary metastases occurring 2–8%^[8]^.

It is found that genes responsible for some key processes such as cellular survival and proliferation (Tp53 and EGFR), cell cycle control (CDKN1A), and cellular differentiation (NOTCH1) are mutated^[9]^. Mostly head and neck SCC spread through the lymphatic route^[10]^. It is thought that hematogenous spread and perineural invasion (PNI) may be related to the brain metastasis of head and neck cancer. PNI can originate from head and neck and may spread through seeding the intrafunicular spaces of the nerve that are in communication with subarachnoid spaces sometimes presenting with neuropathy or obscured by Wallerian degeneration^[^11,12^]^. Moreover previously, laryngeal cancer with PNI was thought to spread via lymphatics within nerves, but this theory was dismissed since lymphatic vessels do not enter the nerve sheath. Now, it is believed that tumor cells travel along nerves through low-resistance pathways, guided by neurotrophic factors like NGF, BDNF, and NT-3 via specific receptors^[13]^.

Screening for distant metastasis in patients with head and neck carcinoma is not yet clearly defined. In existing literature, radiological imaging such as magnetic resonance imaging and computed topography has been widely used. MRI can diagnose PNI better than CT scans. But the FDG-PET/CT is also considered an appropriate test for laryngeal cancer with metastasis^[^14,15^]^.

Due to its rarity, no set standard of management plan exists. So making it difficult to treat and limiting the predictable outcomes of different treatment modalities and prognosis of it. According to the American Joint Committee on cancer TNM staging, T1 and T2 stages must be dealt with surgery and radiotherapy. While in our case (T3 and T4), total laryngectomy with radiotherapy is done. Recently, microlaryngeal surgery has shown great survival rates too^[16]^.

In existing literature to our knowledge, 21 cases of laryngeal carcinoma with brain metastasis exist, in which different treatment modalities have been used. For example, Tressara and Warwick reported cases of laryngeal carcinoma with brain metastasis both given no treatment^[^17,18^]^.

In 2014, Ghosh-Lasker reported 17 cases of brain metastasis secondary to head and neck SCC out of which four originated in the larynx. All patients were treated radically and received radiotherapy to the primary site – either adjuvant or definitive and for brain metastasis all patients received palliative whole brain radiotherapy^[8]^. Nicola Montano et al. reported a case which was treated with chemotherapy plus radiotherapy and for hemiparesis caused by brain metastasis occipital craniotomy and total removal of the tumor^[7]^. In our case particularly, targeted immunotherapy and palliative radiotherapy were given. In existing literature, presenting complaints of epileptic seizures with brain metastasis is not found while being one of the major presentations in our cases. The seizures were controlled by oral antiepileptics after treatment modalities described prior were done.

Brain metastasis is associated with poor prognosis, with median survival typically ranging between 2.3 and 7.1 months^[14]^. Moreover, Mesolella et al. reported that PNI is a poor prognostic factor for laryngeal SCC^[13]^.

As in our case, patient has not been followed up for a long time till now to comment on any recurrence or complications arising post treatment.

This case illustrates an unusual progression of LSCC with brain metastases presenting as new-onset seizures. Early recognition and aggressive supportive management are crucial for optimizing patient outcomes. Further studies are needed to explore effective treatment strategies for LSCC with distant brain metastases.

## Conclusion

Intracranial metastasis from LCSS is exceptionally rare and often presents with nonspecific neurological symptoms, as seen in this case of new-onset seizures. This report highlights the importance of maintaining a high index of suspicion for brain involvement in patients with advanced LSCC presenting with neurological signs. Timely neuroimaging and multidisciplinary management are essential for appropriate diagnosis and symptom control. Although prognosis remains poor, individualized palliative strategies can improve quality of life. Further research is warranted to guide optimal management of such rare metastatic presentations.

## Data Availability

All the relevant data have been included in the manuscript itself.
